# Prevalence and clinical management of cytomegalovirus retinitis in AIDS patients in shanghai, china

**DOI:** 10.1186/1471-2334-11-326

**Published:** 2011-11-24

**Authors:** Ying Shi, Hongzhou Lu, Taiwen He, Yalin Yang, Li Liu, Renfang Zhang, Yufang Zheng, Yinzhong Shen, Yunzhi Zhang, Zhiyong Zhang

**Affiliations:** 1 Shanghai Public Health Clinical Center affiliated to Fudan University, Shanghai 201508, China; 2 Department of Infectious Disease, HuaShan Hospital affiliated to Fudan University, Shanghai 200040, P.R China

**Keywords:** cytomegalovirus retinitis, acquired immunodeficiency syndrome, prevalence, ganciclovir

## Abstract

**Background:**

Cytomegalovirus retinitis is a common AIDS-associated illness, leading to blindness in up to 30% of patients. This study was to investigate the prevalence and clinical management of the cytomegalovirus retinitis associated with AIDS in a large municipality of China.

**Methods:**

Clinical and laboratory data from 23 cytomegalovirus retinitis patients (35 eyes) out of 303 hospitalized AIDS individuals in a single medical center were analyzed retrospectively. Two of 23 patients were diagnosed cytomegalovirus retinitis just before hospitalization without anti-CMV therapy. Ganciclovir combined with the high active anti-retroviral therapy was installed for treatment of cytomegalovirus retinitis after diagnosis was confirmed. The data were analyzed by specialists and statistics was also applied.

**Results:**

The prevalence of cytomegalovirus retinitis in hospitalized AIDS patients was 7.6% in this study. The level of CD_4_^+ ^T lymphocytes was correlated well with the occurrence of cytomegalovirus retinitis, showing 16.8% (19/113) (95% confidence interval: 10.4,25.0), 5.4% (3/56) (95% confidence interval: 1.1,14.9), and 1.4% (1/69) (95% confidence interval: 0.0,7.8) occurrence in the patients with CD_4_^+ ^T lymphocyte counts < 50, 50~99, and 100~199 cells/μl, respectively. The mean CD_4_^+ ^T lymphocyte counts was 31.7 ± 38.6 cells/μl in 23 AIDS patients with cytomegalovirus retinitis. Median CD_4_^+ ^T lymphocyte count is 20 cells/μl with inter-quartile range as (5, 36). Seven patients died (11 eyes) and 16 patients (24 eyes) survived. The proportion of blindness and low vision in eyes infected with cytomegalovirus retinitis respectively was 20.8% (5/24) and 29.2% (7/24) when they were diagnosed in survivors. The ganciclovir therapy was effective in 16 patients (24 eyes). Clinical recovery of cytomegalovirus retinitis was 41.7% (10/24) and clinical improvement 58.3% (14/24). After anti-CMV treatment, the proportion of blindness or low vision was 16.7% (4/24).

**Conclusions:**

The AIDS patients with CD_4_^+ ^T lymphocyte < 50 cells/μl had increased susceptibility to cytomegalovirus associated retinitis. Cytomegalovirus retinitis is a serious disease causing blindness. The cytomegalovirus retinitis in the AIDS patients was response well to ganciclovir therapy. We should check their eyes routinely such as dilated fundus examination with an indirect ophthalmoscope in the AIDS patients with CD_4_^+ ^T lymphocyte counts < 50 cells/μl.

## Background

Cytomegalovirus (CMV) is the largest genome of the herpes viruses. In Shenzhen, China, CMV IgG positive rate in volunteering blood donor is 98.5%[1]. People with normal immune systems rarely develop clinical symptoms or long-term sequelae after initial infection. This latent status remains for their lifetime unless individual suffers from a significant local or systemic immunodeficiency, such as corticosteroid therapy, pharmacologic immunosuppression, autoimmune disorders and acquired immunodeficiency syndrome (AIDS). End-organ disease caused by CMV occurs among persons with advanced immunosuppression, typically those with CD_4_^+ ^T lymphocyte counts <50 cells/μl who are either not receiving or have failed to respond to active antiretroviral therapy (ART). Other risk factors include previous opportunistic infections (OI) and high plasma human immunodeficiency virus (HIV) RNA levels (>100,000 copies/ml). In general, 25 to 42% of AIDS patients in developed countries developed CMV retinitis before introduce of ART and the incidence of CMV retinitis has been decreased 55-95% in the ART era[2]. The incidence of CMV retinitis in India varied from 2 to 20% with the majority of patients receiving ART[3] while the incidence of CMV retinitis in sub-Saharan Africa varied from 0 to 19.6%[4,5]. It was also reported that 19.8% of ART-treated patients developed CMV retinitis in Thailand[6]. The variation of the incidence of the CMV retinitis in developing countries could result from access of diagnostics and health care funding, varied ART regimes and standards in those countries, immune status and genetic susceptibility of the AIDS patients. Despite the ART has changed the incidence and severity of CMV retinitis of AIDS patients, CMV retinitis remains the most common cause for vision loss in AIDS patients globally. Nowadays, 90% of HIV-infected people live in the developing countries including Southeast Asia. The prevalence of AIDS in China has increased significantly for past decade. The case numbers of HIV-infected patients increased 153.0% and of diagnosed AIDS patients increased 209.9% from 2004 to 2007, reaching 38,201 and 9,781 cases, respectively[7]. However, there is the lack of epidemiological report on the incidence of CMV retinitis in the AIDS patients in China. In this retrospective study, CMV retinitis in 303 AIDS patients hospitalized in a single health center specialized for infectious diseases was analyzed, including the prevalence, relationship between CMV retinitis and CD_4_^+ ^T lymphocyte counts, CMV viral load in plasma, clinical treatment regime and response to anti-CMV therapy. This data reflects the incidence of CMV retinitis in AIDS patients and current status of clinical management on this opportunistic infection in China.

## Methods

### Study population

Total of 303 hospitalized AIDS patients (3 or 4 stage of WHO classification, ART naive) were enrolled in this study from October 2005 to December 2007 in Shanghai Public Health Clinical Center. All the patients were examined their eyes routinely and serum samples were taken after they had been admitted to this hospital. Their clinical information (age, mode of transmission, CD_4_^+ ^T lymphocyte count, CMV viral load, CMV IgG, CMV IgM, HIV viral load, examination of eyes) was collected from medical record under their writing consent (Table 1).

**Table 1:** The clinical data of CMV retinitis patients with AIDS before ganciclovir therapy and after ganciclovir therapy

Patient	Age	Gender	CD_4_^+^	Mode	Other	HIV VL	Log-HIV	CMV-	CMV-	CMV VL	Log-CMV	Vision Acuity
ID	(years)		Count	of	OI	(copies ml)		IgM	IgG	(copies/ml)		
			(cells/μl)	transmission								
1 (bi)	55	M	157	S	TB	1.78 × 10^3^	7.48	Neg	Pos	2.37 × 10^3^	7.77	20/25(R), 20/32(L)
2	34	M	4	B	PJP	3.60 × 10^5^	12.79	Pos	Pos	Neg	NA	20/32(L)
3	46	F	20	B	TB	1.21 × 10^5^	11.7	Neg	Pos	Neg	NA	20/50(L)
4	37	M	6	S	OFI,PFI	2.16 × 10^5^	12.28	Neg	Pos	6.24 × 10^3^	8.74	20/63(L)
5 (bi)	33	M	53	B	PFI	5.11 × 10^2^	6.24	Neg	Pos	3.65 × 10^3^	8.20	20/63(R), 20/200(L)
6	34	M	99	B	N	1.24 × 10^3^	7.12	Neg	Pos	5.75 × 10^3^	8.66	20/250(R)
7 (bi)	42	M	7	S	HZ,PJP,	1.71 × 10^4^	9.75	Neg	Pos	8.57 × 10^3^	9.06	20/200(R), 20/100(L)
8	52	M	45	U	TB	6.10 × 10^5^	13.32	Neg	Pos	2.51 × 10^3^	7.83	20/25(R)
9 (bi)	26	M	28	S	PC	7.74 × 10^4^	11.26	Neg	Pos	1.87 × 10^4^	9.84	20/50(R), 20/50(L)
10	48	M	5	U	PJP	1.67 × 10^4^	9.72	Neg	Pos	1.23 × 10^5^	11.72	20/50(R)
11 (bi)	25	F	3	S	N	1.80 × 10^5^	12.1	Neg	Pos	Neg	NA	20/200(R), 20/20(L)
12	35	M	28	S	L,PI	3.87 × 10^4^	10.56	Neg	Pos	3.34 × 10^5^	12.72	20/400(L)
13 (bi)	42	M	1	U	FI	1.10 × 10^6^	13.91	Neg	Pos	1.08 × 10^5^	11.59	20/50(R), HM/BE(L)
14 (bi)	34	M	97	S	CM	3.81 × 10^4^	10.55	Neg	Pos	8.08 × 10^3^	9.00	20/100(R), HM/BE(L)
15	26	M	36	S	N	6.55 × 10^5^	13.39	Neg	Pos	2.04 × 10^3^	7.62	HM/BE(L)
16 (bi)	25	M	5	S	CMVM	2.56 × 10^4^	10.15	Neg	Pos	3.86 × 10^3^	8.26	LP (bi)
17 (bi)	33	F	3	S	CNSI	4.91 × 10^4^	10.8	Neg	Pos	3.37 × 10^4^	10.43	20/63(R), 20/100(L)
18	73	F	17	S	PJP	6.64 × 10^5^	13.41	Neg	Pos	1.31 × 10^3^	7.18	20/200(L)
19 (bi)	59	M	16	S	PJP	1.89 × 10^5^	12.15	Neg	Pos	6.50 × 10^6^	15.69	20/200(R), 20/100(L)
20 (bi)	42	M	33	S	FI	2.06 × 10^3^	7.63	Neg	Pos	1.69 × 10^6^	14.34	20/200(R), HM/BE(L)
21	30	M	0	S	PJP	2.73 × 10^5^	12.52	Neg	Pos	3.64 × 10^5^	12.80	HM/BE(R)
22 (bi)	73	M	35	S	PJP	6.64 × 10^5^	13.41	Neg	Pos	2.03 × 10^5^	12.22	NA
23	59	M	30	S	TB	2.50 × 10^4^	10.13	Neg	Pos	1.78 × 10^5^	12.09	20/40(R)

**Patient**	**Time**	**Time**		**response to anti-CMV**		**treatment/**	**CMV VL**	CD_4_^+ ^Counts*	Vision Acuity*			
**ID**	**HAART Received**	**Anti-CMV Received**		**treatment**		**complications**	**(copies/ml)**	**(cells/μl)**				
	**(weeks)**	**(weeks)**										

1 (bi)	72	4		Effective		NO	Neg	235	20/20(R), 20/20(L)			
2	24	24		Effective		NO	Neg	58	20/20(L)			
3	28	24		Effective		NO	Neg	248	20/20(L)			
4	16	8		Effective		IRU	Neg	165	20/32(L)			
5 (bi)	40	28		Effective		NO	Neg	227	20/20(R), 20/25(L)			
6	24	24		Effective		NO	Neg	400	20/20(R)			
7 (bi)	4	3		Effective		IRU	Neg	186	20/20(R), 20/20(L)			
8	4	2		Effective		NO	Neg	460	20/20(R)			
9 (bi)	12	6		Effective		NO	Neg	177	20/32(R), 20/32(L)			
10	8	2		Effective		NO	Neg	166	20/32(R)			
11 (bi)	8	8		Effective		NO	Neg	120	20/20(R), 20/20(L)			
12	28	2		Effective		NO	Neg	121	20/32(L)			
13 (bi)	20	2		Effective		NO	Neg	189	20/25(R), 20/400(L)			
14 (bi)	12	12		Effective		NO	Neg	Missing	20/32(R),CF/50 cm(L)			
15	24	16		Effective		Rel, RD	Neg	182	20/50(L)			
16 (bi)	20	8		Effective		NO	Neg	222	HM/BE(R),CF/BE(L)			
17 (bi)	0*	0*		NA		NA	NA	NA	NA			
18	0*	0*		NA		NA	NA	NA	NA			
19 (bi)	2	1.3		NA		NA	NA	NA	NA			
20 (bi)	6	0*		NA		NA	NA	NA	NA			
21	8	0*		NA		NA	NA	NA	NA			
22 (bi)	0*	0*		NA		NA	NA	NA	NA			
23	0*	0*		NA		NA	NA	NA	NA			

CD_4_^+ ^count: CD_4_^+ ^count when diagnosed CMV retinitis; CD_4_^+ ^count*: CD_4_^+ ^count when follow-up finished; Vision Acuity: vision acuity when diagnosed CMV retinitis; Vision Acuity*: vision acuity when follow-up finished; HM/BE: hand movement/before eye; LP: light perception; NA: not available; bi: bilatéral; HIV VL: HIV viral load; CMV VL: CMV viral load; F: female; M: male; S: sexually transmission; B: blood infusion; U: unknown; Other OI: other opportunistic infection; TB: tuberculosis; PJP: pneumocystis jiroveci pneumonia; OFI: oral fungal infection; PFI: pulmonary fungal infection; N: no other opportunistic infection but CMV retinitis; HZ: herpes zoster; PC: pancreatitis; L: lymphoma; PI: pulmonary infection; FI: fungal infection; CM: cryptococcal meningitis; CMVM: CMV myelitis; CNSI: central nervous system infection; Time ART received: the time of ART from beginning ART to ending the follow-up; Time Anti-CMV received: the time during anti-CMV treatment with ganciclovir; bi: bilateral; HIV VL: HIV viral load; CMV VL: CMV viral load; Log-HIV: Log-transform of HIV viral load; Log-CMV: Log-transform of CMV viral load; CF/50 cm: count finger/50 cm; HM/BE: hand movement/before eye; CF/BE: count finger/before eye; NA: not available; 0* indicated that the therapeutic regime was not started or less than one week and the patients were died shortly after admitted to the hospital; Rel: relapse; RD: retinal detachment; NO: no ocular complications

This study was a retrospective non-blind study conformed to the tenets of the Declaration of Helsinki. It was approved by Institutional Ethics Review Board of Shanghai Public Health Clinical Center (International index IORG0006364).

### Diagnosis of CMV retinitis

The CMV retinitis was diagnosed by experienced ophthalmologists according to the medical history, ophthalmoscopic appearance of typical retinopathy (yellow-white retinal lesions with granular border or arciform retinal lesion with or without hemorrhage along with vessels[8]) and laboratory assessment of immune status excluded HIV retinopathy, toxoplasmosis retinitis, acute retinal necrosis, progressive outer retinal necrosis, syphilitic retinochoroiditis. After receiving ART, immunologic reconstitution uveitis (IRU) may happen in some patients. IRU is defined as the occurrence of a decrease in vision because of moderate or severe vitritis, macular edema, epiretinal membranes, keratic precipitate, neovascularization of the optic disk or retina, posterior synechia and cataract in patients whose CD_4_^+ ^T lymphocyte count has increased after receiving ART[9]. The examinations were performed including vision acuity examination (The Snellen Eye Chart is read while standing 20 feet from the chart.), the slit-lamp ophthalmoscope, indirect ophthalmoscope (dilated fundus examination), fundus photograph (taken to document progression or regression). The level of CMV IgG and IgM and CMV viral load were analyzed by ELISA and real-time quantitative PCR respectively. The CD_4_^+ ^T lymphocyte count was done using the flowcytometry. HIV viral load was also quantified using bDNA technology in all patients. The diagnostic methods were compliant with the standard of the AIDS Group of Society of Infectious Diseases, Chinese Medical Association[10]. Twenty-three patients were confirmed CMV retinitis (Table 1) and they were followed up once a week. Follow-up continued for at least 6 months. If the AIDS patients were discharged from hospital, they were followed up in out-patient department.

### Treatment of the CMV retinitis patients with AIDS

The ganciclovir therapy was installed for the patients with CMV retinitis in combination with ART. Twenty-one patients with CMV retinitis had received the ART treatment. The other 2 CMV retinitis patients with AIDS died soon, so there was not enough time to treat them with ART. Ganciclovir (5 mg/kg) was administered twice a day by intravenous injection for two to three weeks (according to the severity) during the inductive phase. Then ganciclovir (5 mg/kg) was given once a day intravenously or ganciclovir (1000 mg) three times a day orally during the maintenance phase. The therapy of ganciclovir should be continued until the CMV retinitis is completely inactive, the CD_4_^+ ^T lymphocyte counts above 75-150 cells/μl at least 3 months, and the patient on ART for 18 months[11].

### Statistical analysis

Statistical analyses were conducted using SAS statistical Software. Different prevalences of CMV retinitis between 4 groups of AIDS patients according to different levels of CD_4_^+ ^T lymphocyte counts were analyzed using the Cochran-Armitage trend test. *P *values < 0.05 is considered to be a significant level.

## Result

### Prevalence of the CMV retinitis

Three hundred and three patients were all in clinical stage 3 or 4 according to WHO disease staging system for HIV infection (Table 2). The average age of patients was 41.9 ±14.3 years, ranging from 25 to 73 years. Twenty-three patients with CMV retinitis were diagnosed among 303 AIDS patients, 19 males (29 eyes) and 4 females (6 eyes). Two of 23 patients were diagnosed CMV retinitis just before hospitalization without anti-CMV therapy. In 23 patients with CMV retinitis, 16 patients acquired their AIDS via sexually transmission (16/23 = 69.6%), 4 via blood infusion (4/23 = 17.4%) and 3 with unknown history (3/23 = 13.0%). Unilateral CMV retinitis was seen in 11 patients and bilateral in 12 patients. Other opportunistic infections in the cohort of 23 AIDS patients were tuberculosis, pneumocystis jiroveci pneumonia, oral fungal infection, pulmonary fungal infection, herpes zoster, lymphoma, cryptococcal meningitis, CMV myelitis, central nervous system infection (Table 1).

**Table 2:** The occurrence of CMV retinitis in 303 AIDS patients grouped by their CD_4_^+ ^T lymphocyte counts

No. of CD_4_^+^ T cells (cells/μl)	No. of cases	No. of CMV retinitis patients	CMV retinitis prevalence (95% CI) (%)
<50	113	19	16.8 (10.4,25.0)
50~99	56	3	5.4 (1.1,14.9)
100~199	69	1	1.4 (0.0,7.8)
200~350	65	0	0 (0.0,5.5)

95% CI: 95% confidence interval

The prevalence of CMV retinitis was 7.6% (23/303) in this cohort study with 303 AIDS patients. No CMV retinitis was observed in 65 patients with CD_4_^+ ^T lymphocyte counts between 350 to 200 cells/μl and one CMV retinitis out of 69 patients with CD_4_^+ ^T lymphocyte counts between 199 to 100 cells/μl (prevalence 1.4%, 95% confidence interval: 0.0,7.8). The CMV retinitis occurred in 3 out of 56 patients with CD_4_^+ ^T lymphocyte counts at level of 99 to 50 cells/μl (prevalence 5.4%, 95% confidence interval: 1.1, 14.9). Moreover, the CMV retinitis was diagnosed in 19 out of 113 patients with CD_4_^+ ^T lymphocyte counts < 50 cells/μl (prevalence 16.8%, 95% confidence interval: 10.4, 25.0) (Table 2). Statistical analysis showed a trend that the prevalence of CMV retinitis was significantly difference in the AIDS patients with different levels of the CD_4_^+ ^T lymphocyte counts (p < 0.001, Cochran-Armitage trend test).

### Clinical manifestations pre- and post- HAART and anti-CMV treatment

**Figure 1:**
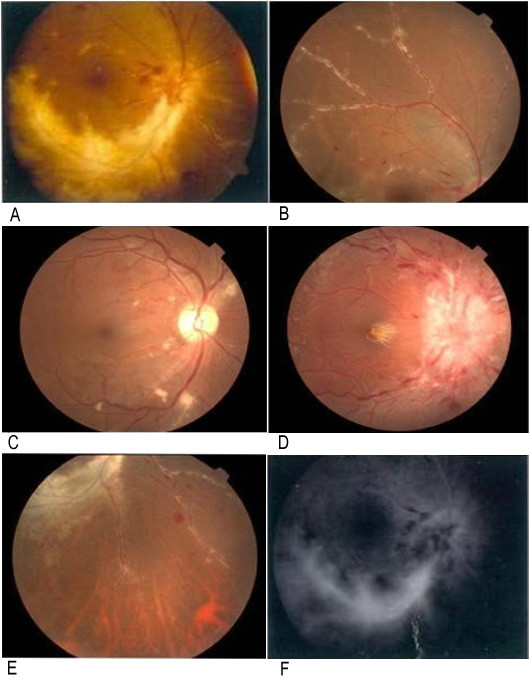
**Typical retinal appearance of the patients with CMV retinitis**. A yellow-white retinal necrosis with granulo-margin. B retinal frosted branch angiitis. C white cotton-wool patches. D optic disc edema. E sclerosis and occlusion of retinal vessels. F fluorescence leakage by fluorescent fundus angiography.

Twenty-three patients (35 eyes) suffered from incomplete loss of vision acuity to blindness during enrollment of the study. In 23 CMV retinitis patients (35 eyes), the vision acuity less than 20/400 defined blindness in 7 eyes and between 20/400 and 20/63 defined low vision in 15 eyes. Yellow-white retinal lesions with granular border were observed. There were 4 eyes with optic neuritis and disc edema and retinal frosted branch angiitis in 7 eyes (Figure 1). Sclerosis and occlusion of retinal vessels occurred at the later stage of CMV retinitis. Keratic precipitates existed in 2 of 35 eyes by slit-lamp microscopic examination.

Eighteen patients with the CMV retinitis had received the ART and ganciclovir, 3 patients the ART without ganciclovir and 2 patients neither ART nor ganciclovir. Seven AIDS patients with CMV retinitis in 11 eyes (include 3 patients without ganciclovir therapy and 2 patients without ganciclovir therapy and ART) died within 2 weeks of enrollment because of systemic complications and infections. Sixteen patients (24 eyes) had clinical follow-up data available after varied duration of anti-CMV treatment. The proportion of blindness and low vision in eyes infected with CMV retinitis respectively was 20.8% (5/24) and 29.2% (7/24) when they were diagnosed in survivors. Clinical manifestations of CMV retinitis had altered after treatment comparing to clinical signs pre-treatment. In recovered patients, hemorrhage and exudation were disappeared completely and yellow-white retinal scars were formed in 10 eyes. The visions in 23 of 24 eyes improved (95.8%) but the visual acuity in 3 eyes was still lower than 20/400 and in 1 eye was 20/400. The proportion of blindness or low vision was 16.7% (4/24) after anti-CMV treatment. IRU (keratic precipitate and vitritis) occurred in 3 eyes (2 patients) out of 24 eyes (12.5%) after 8 to 20 weeks of combined ART with anti-CMV treatment, Detachment of retina was also observed in 1 out of 24 eyes (4.2%) after discontinuation of the therapy. The CMV retinitis was relapsed in one patient. The patient continued ART for 6 weeks when he relapsed, but he was still immunosuppressed.

### Laboratory Evaluation

The average of HIV viral DNA load was 2.3 ± 3.0 × 10^5 ^copies/ml (median of 7.7 × 10^4^) in all AIDS patients with CMV retinitis enrolled into this study. Further analysis on the log-transformation of this characteristic derives a mean of 11.0 ± 2.2 for these twenty-three patient cohort.

The mean CD_4_^+ ^T lymphocyte count was 31.7 ± 38.6 cells/μl in the AIDS patients with CMV retinitis. Median CD_4_^+ ^T lymphocyte count is 20 cells/μl with inter-quartile range as (5, 36). Two IRU patients showed an average CD_4_^+ ^T lymphocyte counts of 112 cells/μl when the diagnosis was confirmed, indicating reconstitution of immune capacity after a combined ART with anti-CMV treatment.

The average of CMV viral load at the study enrollment was 4.8 ± 14.7 ×10^5 ^copies/ml in the AIDS patients with retinitis. Further analysis on the log-transformation of this characteristic derives a mean of 10.3 ± 2.5 for these 23 patients cohort.

The CMV-IgM in serum was negative in all but one patient and CMV-IgG was positive in all patients with CMV retinitis.

Clinical and laboratory data from 23 patients with CMV retinitis before and after treatment were summarized in Table 1.

## Discussion

The CMV retinitis becomes one of the most prevalent opportunistic infection in AIDS patients causing blindness. The prevalence of CMV retinitis in the cohort of 303 hospitalized AIDS patients was 7.6% in a single health center in Shanghai, China. It seemed that the occurrence of CMV retinitis in this retrospective study was lower than the prevalence reported in literatures[12]. This prevalence may be over-estimated because the limitation of subjects in our study. But we could find the trend of CMV retinitis of patients in late stage of AIDS. In our understanding from a point of view of Chinese culture on their health and cost concern about doctor's visits, minor vision problem was and will not be the main reason for the AIDS patients to seek medical attention in comparison with other vital signs and symptoms at the later stage of disease course. Some patients only visited the doctor when their diseases were very serious. So many of them died from other infections or complications before CMV retinitis had been diagnosed. For example, seven advanced AIDS patients entered into the study died within 2 weeks after their primary admission to the hospital. Currently there is no well-organized epidemiological survey or study on the prevalence of CMV retinitis in the AIDS patients in China. So the prevalence of CMV retinitis in whole population of AIDS patients with different stages of disease in China still need to be identified. The results from the study indicated the prevalence of CMV retinitis of AIDS patients when they came to hospital for medical treatment. This study raises our awareness of special attention to full examination of the entire retina through a full dilated pupil using an indirect ophthalmoscope of AIDS patients in their first clinical visit since a majority of CMV retinitis could be asymptomatic and diagnosed only through clinical screening and ophthalmologic examination[8]. The study also implies the importance and urgency to have national-wide epidemiology survey on the prevalence of CMV retinitis and to launch educational programs for AIDS patients to gain basic knowledge on retinitis and other potential opportunistic infection in their lifetime.

Seven patients died (11 eyes) and 16 patients (24 eyes) survived. The proportion of blindness and low vision in eyes infected with CMV retinitis respectively was 20.8% (5/24) and 29.2% (7/24) when they were diagnosed in survivors. The ganciclovir therapy was effective in 16 patients (24 eyes). After anti-CMV treatment, the proportion of blindness or low vision was 16.7% (4/24). The rate of blindness and low vision was high. CMV retinitis is a serious disease causing blindness, but the vision acuity of many patients will improve if anti-CMV therapy provided promptly. The vision acuity after anti-CMV in our study is the vision acuity while ending follow-up, so there was limitation. But these results of vision acuity showed the anti-CMV was effective.

Cotton wool patches are often observed in the early stage[13]. It is important to keep in mind that it was often difficult to distinguish the cotton wool patches or hemorrhage along with retinal vessels in the early stage of CMV retinitis from the retinopathy induced by HIV infection. The white cotton wool patches identified in 3 eyes during the initial diagnosis had evolved into large area of yellow-white retinal lesions two months later without treatment of anti-CMV therapy. It becomes clear that early retinopathy can not be ignored and should follow-up closely in the ADIS patients.

According with previous studies[14-16], our result showed that prevalence of CMV retinitis was closely related to the levels of CD_4_^+ ^T lymphocyte counts in 303 AIDS patients in this cohort study. A routine ocular examination should be performed in the AIDS patients with CD_4_^+ ^T lymphocyte counts less than 50 cells/μl. Taken together, the number of CD_4_^+ ^T lymphocyte in peripheral blood is an empirical indicator on occurrence, progress, alleviation and regression of CMV retinitis in clinical diagnostic and management. In our study, CMV retinitis in 4 patients with CD_4_^+ ^T lymphocyte counts > 50 cells/μl were diagnosed without ART. We should observe more cases to study the reason about CMV retinitis diagnosed in high CD_4_^+ ^T lymphocyte counts patients such as race of people, age, complicating other disease et al.

CMV viral load was quantified with the real-time PCR assay to explore whether the level of CMV viral load in blood was correlated to the severity of CMV retinitis in this study. There was limitation to detect the level of CMV viral load in blood, sometimes CMV in the blood does not rule out the possibility of a different infection in the eye. It was reported that the CMV-PCR assay had a high predicting value with estimated sensitivity of 95% in detecting untreated CMV retinitis if vitreous samples were used[17]. However, the vitreous sampling procedure has a risk to cause hemorrhage, infection and vision acuity loss, and was difficult to obtain patient's consent. So vitreous sampling is reserved for unusual cases with atypical features, where clinical findings are ambiguous and there needs to be clarification between other retinitis.

The choice of available drugs includes ganciclovir, foscarnet, cidofovir and fomivirsen. Because of cost and supply issues of anti-CMV drugs, ganciclovir is still the first-line anti-CMV drug for clinical management of CMV retinitis associated with AIDS in this study. In combination with antiretroviral medication, suppression of CMV disease for a short-term and maintenance of vision for a relatively long-term were achieved from this study. The CMV viral loads in patients after anti-CMV therapy were decreased below detectable level. Although it is reported that intravitreal injection of ganciclovir or intraocular implant of sustain-release ganciclovir improved treatment efficacy and reduce side-effect of the drug, this therapeutic procedure has neither been applied in the study nor reported in other central hospitals in China for treatment of CMV retinitis.

IRU is recognized as one of important causes of visions loss[18,19]. Two cases of IRU (3 eyes) were diagnosed in our study (2/23). Their visions were 20/32, 20/40, 20/40, respectively, and average CD_4_^+ ^T lymphocyte count was 112 cells/μl while vitritis and uveitis were observed. Goldberg reported that use of cidofovir increased the risk of IRU by a factor of 3.3[20]. Although immune reaction to low level of CMV antigen in infected eye is essential to IRU pathogenesis, its mechanism is still not fully understood[18].

## Conclusion

Even though authors acknowledged that the case numbers were relatively small in this cohort, it did illustrate occurrence of CMV retinitis in advanced AIDS patients in China. The occurrence of CMV retinitis was reversely correlated with the level of CD_4_^+ ^T lymphocytes. The CMV retinitis in the AIDS patients was response well to ganciclovir therapy. We should check their eyes routinely such as dilated fundus examination with an indirect ophthalmoscope in the AIDS patients with CD_4_^+ ^T lymphocyte counts< 50 cells/μl. In this study, CD_4_^+ ^T lymphocyte counts in 4 patients with CMV retinitis were > 50 cells/μl (157 cells/μl, 53 cells/μl, 99 cells/μl, 97 cells/μl separately). It would be best to check AIDS patients with CD_4_^+ ^T lymphocyte counts < 200 cells/μl. Several important factors such as the result of CMV detection and CD_4_^+ ^T lymphocyte counts have been identified for early clinical diagnosis of CMV retinitis when CMV retinitis is not typical and prophylactic treatment of anti-CMV drug is recommended when the CD_4_^+ ^T lymphocyte count is less than 50 cells/μl in AIDS patients. This study also addressed the necessity and urgency of a national wide epidemiological investigation on CMV retinitis in AIDS patients in China.

## Competing interests

The authors declare that they have no competing interests.

## Authors' contributions

YingS, HL and ZZ conceived of the study, participated in its design and draft the manuscript. THand YY carried out the molecular genetic studies, participated in the PCR detection and drafted the manuscript. LL, RZ, YufangZ, YinzhongS and YunzhiZ participated in data collection. All authors read and approved the final manuscript.

## Pre-publication history

The pre-publication history for this paper can be accessed here:

http://www.biomedcentral.com/1471-2334/11/326/prepub
